# The past, the present, and the future of preclinical mouse models for Alzheimer's disease and related dementias

**DOI:** 10.1002/alz.71741

**Published:** 2026-08-08

**Authors:** Adrian L. Oblak, Michael Sasner, Gregory W. Carter, Gareth R. Howell, Stacey J. Sukoff Rizzo, Karina Leal, Paul R. Territo, Bruce T. Lamb

**Affiliations:** ^1^ Indiana University School of Medicine Indianapolis Indiana USA; ^2^ Stark Neurosciences Research Institute Indianapolis Indiana USA; ^3^ Department of Radiology and Imaging Sciences, Indiana University School of Medicine Indianapolis Indiana USA; ^4^ The Jackson Laboratory Bar Harbor Maine USA; ^5^ The Jackson Laboratory for Genomic Medicine Farmington Connecticut USA; ^6^ Department of Neurobiology University of Pittsburgh School of Medicine Pittsburgh Pennsylvania USA; ^7^ Aging Institute University of Pittsburgh School of Medicine Pittsburgh Pennsylvania USA; ^8^ Sage Bionetworks Seattle Washington USA; ^9^ Department of Medicine, Indiana University School of Medicine Indianapolis Indiana USA; ^10^ Department of Medical and Molecular Genomics, Indiana University School of Medicine Indianapolis Indiana USA

**Keywords:** biomarkers, brain multi‐omics, longitudinal phenotyping, neurodegeneration, precision medicine, preclinical models, translational neuroscience

## Abstract

Over the past decade, the Model Organism Development and Evaluation for Late‐Onset Alzheimer's Disease (MODEL‐AD) consortium has transformed preclinical Alzheimer's disease (AD) research by addressing critical limitations in traditional mouse models that failed to translate to human disease. By leveraging human genetic discoveries, MODEL‐AD has developed > 70 genetically informed mouse models, standardized phenotyping pipelines, and an open‐access data infrastructure aligned with late‐onset AD biology. These models incorporate human risk variants, environmental factors, and aging to better capture disease complexity, including emerging recognition of mixed pathologies such as vascular contributions, Lewy body disease, and TDP‐43 proteinopathy. Despite substantial progress, key challenges remain, including modeling multimorbidity, integrating aging, and improving translational predictability. Here, we outline a strategic roadmap spanning short‐, intermediate‐, and long‐term approaches to refine disease modeling, enhance preclinical testing rigor, and support precision medicine. Continued investment in MODEL‐AD will be essential to accelerate therapeutic discovery and establish a scalable framework for studying complex neurodegenerative diseases.

## Highlights

- Longitudinal multimodal profiling was used to map Alzheimer's disease trajectories in mouse models sampled every three months from 3 to 18 months of age.
- Brain multi‐omics integrated RNA‐based subtyping with proteomic analyses, while prioritization at 12 months identified the most translationally relevant models.
- Deep phenotyping of these models provides a robust foundation for preclinical therapeutic testing.

## 1 INTRODUCTION

In the last decade, MODEL‐AD (Model Organism Development and Evaluation for Late‐Onset Alzheimer's Disease) has emerged as a cornerstone infrastructure for preclinical Alzheimer's disease (AD) research. Created in response to longstanding challenges in modeling late‐onset AD (LOAD), the IU/JAX/PITT MODEL‐AD Center comprises investigators from Indiana University (IU), The Jackson Laboratory (JAX), and the University of Pittsburgh (PITT), has delivered novel, genetically informed mouse models, a translationally relevant phenotyping pipeline, and a rigorous preclinical testing platform. This white paper reflects on the origins, accomplishments, and future trajectory of MODEL‐AD, and articulates why continued investment is both warranted and necessary. It is intended to support strategic discussions with funding agencies, guide future priorities, and serve as a clear articulation of the evolving mission of MODEL‐AD at a pivotal moment in the translational AD research landscape.

### 1.1 MODEL‐AD was created to improve preclinical resources for LOAD

By 2015, AD research had reached a breaking point. Nearly every drug candidate that showed promise in mouse models had failed in human trials. The core issue was poor translational relevance. Most existing models were built around transgenically expressed rare familial AD mutations, which are not representative of most patients with LOAD. These models failed to reflect the complexity and heterogeneity of the disease, often lacked thorough characterization, and produced inconsistent results across laboratories. Compounding the problem, there was no standardized pipeline for phenotyping or testing compounds in vivo, leaving the field without a reliable framework for validating therapeutic targets. At the same time, advances in human genetics were reshaping our understanding of LOAD. Large‐scale genome‐wide association studies (GWASs) and transcriptomic analyses were uncovering dozens of risk genes and molecular pathways linked to the disease. Yet these discoveries remained largely disconnected from model development. The insights weren't being translated into experimental systems that could support drug discovery or mechanistic research.

In response, the National Institute on Aging (NIA) launched the MODEL‐AD program in 2016. Its mission was to address the translational gap by developing genetically relevant models based on human LOAD risk, implementing standardized and reproducible phenotyping pipelines, building infrastructure for rigorous and unbiased therapeutic testing, and making all tools and data openly accessible to the research community (https://adknowledgeportal.synapse.org/). Since its launch, MODEL‐AD has fundamentally reshaped preclinical AD research. In less than a decade, the program has produced > 70 novel mouse models (https://www.model‐ad.org/data‐and‐resources/) that incorporate human genetic variants linked to LOAD. Key human genes that differ significantly between humans and mice (e.g., apolipoprotein E [*APOE*], microtubule‐associated protein tau [*MAPT*]) were introduced into the equivalent mouse gene locus by gene replacement (GR) strategies.^1^ Further, genetic risk variants (coding and non‐coding) that could be readily mapped to the equivalent mouse gene were engineered into mice using CRISPR/Cas9 technology. The program has developed both “base” models (consisting of, for instance, human *MAPT*, *APOE* ε4, and humanized amyloid beta [Aβ] sequence),^2^, ^3^ as well as combinatorial models (incorporating variation in key risk genes such as triggering receptor expressed on myeloid cells 2 [*TREM2*], 1‐phosphatidylinositol‐4,5‐bisphosphate phosphodiesterase gamma‐2 [*PLCG2*], ATP‐binding cassette sub‐family A member 7 [*ABCA7*], methylenetetrahydrofolate reductase [*MTHFR*], interleukin 1 receptor accessory protein [*IL1RAP*], etc.) to better reflect polygenic risk, aligning preclinical systems more closely with human disease biology. In some cases, models have been improved by the incorporation of environmental risk factors (e.g., high‐fat/high‐sugar diet^3^, ^4^) and prioritized models have been assessed from young to older ages.

Intriguingly, even though current models lack hallmark amyloid and tau pathologies, many show features of LOAD including relevant transcriptomic and proteomic changes, imaging‐based abnormalities, synaptic dysfunction, and learning and memory deficits.^2^, ^3^, ^4^, ^5^ All MODEL‐AD mice are maintained on standardized genetic backgrounds and made available without use restrictions through The Jackson Laboratory. This approach ensures not only scientific rigor and reproducibility but also broad adoption across the research community, including pharma, biotech, and other for‐profit entities. Through this work, MODEL‐AD has established a new foundation for AD research, one built on genetic relevance, standardization, and open science.

MODEL‐AD has relied heavily on close interactions and integration with other AD‐relevant consortia, including Accelerating Medicines Partnership for Alzheimer's Disease (AMP‐AD), Target Enablement to Accelerate Therapy Development for Alzheimer's Disease (TREAT‐AD), Marmosets as Research Models of Alzheimer's Disease (MARMO‐AD), Environmental Toxicant Exposures and Alzheimer's Disease (TOX‐AD), Vascular Contributions to Cognitive Impairment and Dementia Center Without Walls (VCID CWOW), and the recently created cell modeling consortium Microphysiological Systems to Advance Precision Medicine for Alzheimer's Disease and Related Dementias (MPS‐AD). Thanks to the vision of program staff from particularly the NIA, as well as other National Institutes of Health (NIH) institutes, AD‐related consortia are required to work collaboratively, not just within their consortia, but importantly across consortia. This has ensured each consortia benefits immensely and at the earliest opportunity from activities within other consortia. For instance: MODEL‐AD uses data from AMP‐AD to evaluate its models for relevance to human AD; TREAT‐AD is using MODEL‐AD resources to accelerate the characterization and experimental validation of the next generation therapeutic targets for AD; TOX‐AD is using MODEL‐AD base strains to evaluate the effect of toxicant exposure on AD risk; and VCID CWOW is evolving MODEL‐AD strains to better model the complexity of vascular contributions to cognitive decline and dementia (VCID). These interacting consortia are ensuring activities are synergized, maximizing efficiency and resource use; embracing new approach methodologies; and when appropriate, replacing, reducing, and refining the use of animals. However, as we strive for effective therapies for AD, preclinical mouse and other animal models will always be necessary for a plethora of critical reasons including (but not limited to) determining mechanisms by which brain region connectivity breaks down, understanding the contribution of different organ systems (e.g., heart, kidney, liver, blood system), understanding the fundamentals of why aging is the greatest risk factor, and possibly most importantly, to test the safety and efficacy of single and combinatorial therapeutic approaches before moving novel treatments to the clinic.

### 1.2 The ongoing challenges, gaps, and opportunities for the MODEL‐AD program

Despite the substantial progress made by the MODEL‐AD program, significant gaps and opportunities remain. The original mandate for MODEL‐AD was to create and characterize improved preclinical models for LOAD. At the time, AD (including LOAD) was considered the most common form of dementia. However, the past decade has seen paradigm shifts in our understanding of the pathologies present in the brains of LOAD patients. We now know most cases diagnosed as AD show additional neuropathologies alongside the classic hallmark AD pathologies of amyloid plaques and neurofibrillary tangles of tau. Studies suggest “clean” AD represents only 10% of AD cases,^6^ meaning that classical amyloid–tau AD is no longer the most common form of dementia.

Studies show many cases of LOAD include a cerebrovascular deficit. Cerebral amyloid angiopathy (CAA), a form of cerebral small vessel disease (cSVD), has long been associated with LOAD. However, it is now widely accepted that a broader spectrum of vascular‐related pathologies co‐occur with amyloid and tau pathology including cSVD (CAA, arteriolosclerosis), small and large vessel infarcts, white matter hyperintensities (WMHs), and cerebral microhemorrhages, commonly referred to as VCID. There is a strong correlation between peripheral syndromes (e.g., hypertension, obesity) and cerebrovascular deficits, and so VCID is commonly modeled using environmental approaches such as diets high in fat, sugar, cholesterol, and/or salt. In the MODEL‐AD program, in addition to a high‐fat/high‐sugar diet, we have used genetic risk factors to model cSVD such as the 677C > T variant in methylene tetrahydrofolate reductase (MTHFR*677C > *T*).^7^ In addition to human risk factors, alternative inbred mouse strains (e.g., WSB/EiJ) that are more susceptible to vascular related pathologies compared to the commonly used C57BL/6J, have been incorporated into preclinical modeling.^8^

In addition to vascular pathologies, brains of AD patients often include pathologies traditionally used to define other dementias such as Lewy bodies (Parkinson's disease) and TDP‐43 (limbic‐predominant age‐related TDP‐43 encephalopathy [LATE]). These observations have led to classifications of dementia that include AD+, mixed dementia, or mixed etiology dementia (MeD), although standardized terms are yet to be established. One can speculate that given most cases of AD include multiple pathologies, this likely contributes to the limited success of anti‐amyloid therapies. Therefore, it is critical we consider this evolution in our understanding of LOAD and related dementias when creating preclinical models.

### 1.3 Expanding the scope of preclinical mouse models for AD and related dementias

The MODEL‐AD program is addressing the challenges of modeling the emerging complexities of LOAD and related dementias in three distinct phases. As a short‐term approach, to provide improved models for preclinical testing at the earliest opportunity, we are combining familial AD (fAD) risk variants in amyloid precursor protein (*APP*) and *MAPT* knock‐in models. These are a compromise, in that they include genetic risk factors not found in LOAD patients, but they will be significant improvement over the commonly used transgenic models. For example, heterozygous mice for the *APP^SAA^* allele crossed to *MAPT‐* GR models with risk variants would be expected to have late‐onset amyloid and tau pathology, disease‐relevant biomarker signatures, along with concomitant neuropathology and synaptic loss.

In the intermediate term, we are combining our base models expressing necessary genetic elements such as humanized *APP*, *MAPT*, and *APOE* ε4 with common, low‐risk variants associated with LOAD in *PLCG2*, *MTHFR*, sortilin related receptor 1 (*SORL1*), *IL1RAP*, and so on. These are expected to have milder phenotypes with a later age of onset but will most accurately model clinical LOAD. As clinical diagnosis advances to better stratify dementia patients, our goal is to be able to provide distinct models for each classification of dementia type. Long term, we plan to shift to models with combinations of gene replacement alleles with AD‐related human loci knocked in to replace the corresponding mouse locus.^1^ These GR models have the advantages of: expressing human mRNA and protein at endogenous levels, so that preclinical testing can engage human targets (e.g., in vivo editing of human *APP*; antisense oligonucleotides to alter human *TAU/MAPT* splicing; and immunotherapies against human Aβ) and incorporating non‐coding elements that carry significant LOAD genetic risk but are generally not well conserved between human and mouse. We have already made significant progress by combining GR models expressing *APP*, *MAPT*, *APOE* ε4, TAR DNA binding protein (*TARDBP*), synuclein alpha (*SNCA*), and transmembrane protein 106B (*TMEM106B*). Most of these alleles have both a wild‐type and risk version. In addition, we can use these as platform models to introduce new variants by a simple CRISPR process, rather than having to perform extensive breeding to get new models. We are confident that this approach will enable the development of a panel of models with multifactorial pathologies such as Lewy body disease, TDP43 pathology, LATE, and vascular compromise in addition to amyloid and tau pathology.

In all our future work, we will continue to use environmental risk factors (e.g., high‐fat diet) and non‐standard genetic backgrounds to drive more robust AD‐like phenotypes in a human‐relevant manner. These models will be developed and prioritized with a focus on fluid biomarkers that will provide practical measures of disease progression and therapeutic efficacy that can be used in preclinical testing. In all cases, we will continue to critically evaluate sex‐based phenotypic differences.

Another barrier to using LOAD models that are dependent on aging is that both academic and pharma/biotech have intense pressure to move rapidly. Too often in the past, the quest has been to load the mouse brain with pathology(ies) in young mice to provide convenient models for rapid studies. However, aging is the greatest risk factor in dementias and should be considered a key element in preclinical modeling. Age‐related changes in immune activity and metabolic processes make it likely that the aged brain differentially drives and/or responds to neuropathological protein aggregation and may similarly respond differently to treatments. In the MODEL‐AD program to date, aging has been considered a key component of any model. The next generation of mouse models will need to be carefully characterized to understand trajectories of healthy and pathological aging, so that interventions can be tested at the appropriate life stage. One solution to this challenge of incorporating aging into AD studies could be for mouse model suppliers like The Jackson Laboratory to provide cohorts of aged mice so that studies could be initiated at an appropriate therapeutic window as previously defined by model characterization using clinically relevant measures. This approach has proven successful for aging research and could be successful for AD research.

### 1.4 Translationally relevant phenotyping

**FIGURE 1:**
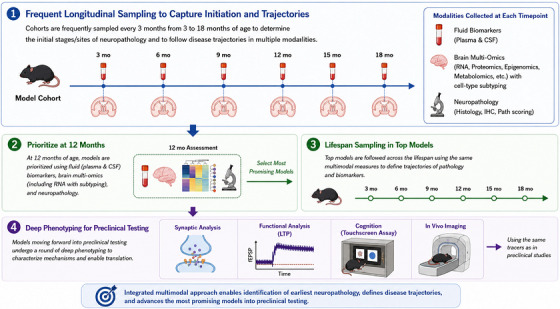
Longitudinal phenotyping and model prioritization pipeline for translational Alzheimer's disease studies. Cohorts are frequently sampled every 3 months from 3 to 24 months of age to identify the earliest stages and anatomical sites of neuropathology and to define disease trajectories across multiple modalities. At 12 months, models are prioritized based on fluid biomarkers (plasma and CSF), brain proteomics, and neuropathological assessments. The most promising models are then followed longitudinally across the lifespan using the same multimodal measures to characterize progression of pathology and biomarker changes. Models selected for preclinical testing undergo deep phenotyping, including synaptic analyses, functional studies such as LTP and cognitive testing, and in vivo imaging using the same tracers planned for therapeutic studies to support translational relevance. Made using BioRender. CSF, cerebrospinal fluid; LTP, long‐term potentiation.

Comprehensive phenotyping of mouse models for preclinical studies must mimic clinical studies as closely as possible. Therefore, we will focus heavily on fluid biomarkers that can be assayed longitudinally with frequent sampling (see Figure 1). In addition, these studies should take advantage of the ability to cross‐sectionally harvest tissues in animals models, to get a detailed understanding of mechanisms of disease progression that is generally not possible in clinical studies. We plan to densely sample cohorts (e.g., every 3 months from 3 to 24 months) to be able to determine the initial stages/sites of neuropathology, and to follow disease trajectories in multiple modalities throughout disease progression. Specifically, we propose to prioritize models at 12 months of age using fluid (plasma and cerebrospinal fluid) biomarkers, brain proteomics, and neuropathology. Those models that look most promising will then be sampled across the lifespan for these measures. For models to move into preclinical testing, they will then undergo a round of deep phenotyping including synaptic analysis, functional analysis (long‐term potentiation and cognition) as well as in vivo imaging using the same tracers to be used in preclinical studies.

To ensure translational validity, neuropathological assessment of MODEL‐AD mouse models will follow standardized protocols that align with human neuropathology practices used across NIA‐funded Alzheimer's Disease Research Centers (ADRCs). These include comprehensive evaluation of core and co‐pathologies such as Aβ plaques (Thal phase), tau neurofibrillary tangles (Braak stage), neuritic plaques (Consortium to Establish a Registry for Alzheimer's Disease score), TDP‐43 inclusions, α‐synuclein Lewy bodies, CAA, vascular lesions (microinfarcts, arteriolosclerosis), and hippocampal sclerosis. In the mouse models, analogous assessments will be performed using immunohistochemical staining and quantitative image analysis in age‐matched cohorts, guided by harmonized criteria such as those outlined in the NIA–Alzheimer's Association guidelines and National Alzheimer's Coordinating Center (NACC) neuropathology data form. By mapping mouse findings to human scoring systems, we aim to calibrate model fidelity, stratify models based on co‐pathology burden, and enhance the predictive power of preclinical findings for therapeutic translation.

### 1.5 What is MODEL‐AD's role in the future of preclinical mouse models for LOAD?

**FIGURE 2:**
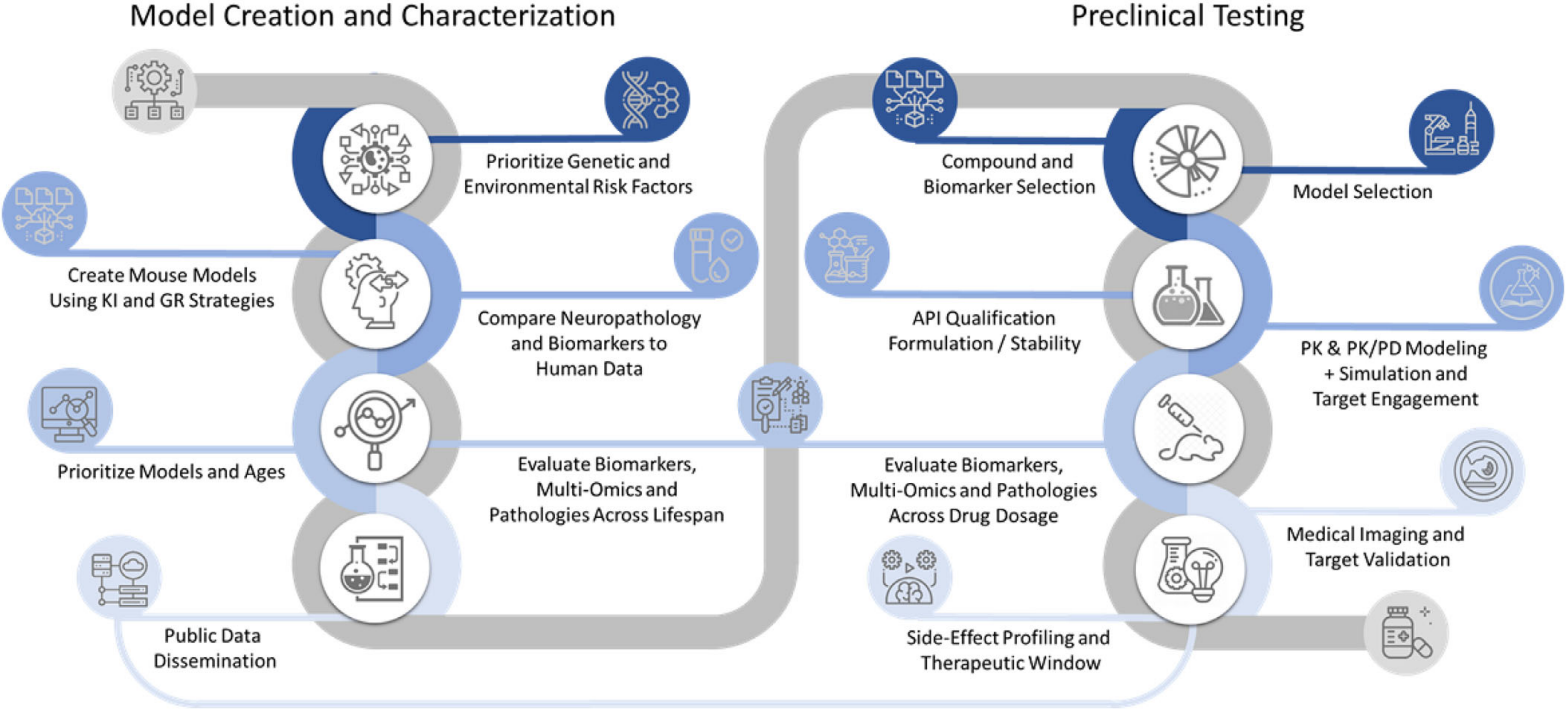
Overview of the MODEL‐AD translational pipeline for developing and evaluating preclinical mouse models of LOAD. This schematic illustrates the end‐to‐end framework established by the MODEL‐AD consortium, integrating model development, phenotyping, and preclinical therapeutic testing. The left panel highlights model generation strategies, including incorporation of human genetic risk variants (e.g., *APOE*, *TREM2*, *MAPT*), gene‐replacement approaches, and environmental modifiers such as diet, all designed to capture the complexity of LOAD. These models are subjected to standardized, longitudinal phenotyping pipelines encompassing multi‐omic profiling, imaging, neuropathology, and functional assessments aligned with clinical measures. The right panel depicts the preclinical testing pipeline, including compound screening, pharmacokinetic/pharmacodynamic evaluation, target engagement, biomarker analysis, and cognitive testing. Iterative feedback between model development and therapeutic testing, supported by open data sharing through the AD Knowledge Portal, enables continuous refinement of model fidelity and translational relevance, advancing precision medicine approaches for Alzheimer's disease and related dementias. API, active pharmaceutical ingredient; *APOE*, apolipoprotein E; GR, gene replacement; KI, knock‐in; LOAD, late‐onset Alzheimer's disease; *MAPT*, microtubule‐associated protein tau; MODEL‐AD, Model Organism Development and Evaluation for Late‐Onset Alzheimer's Disease; PD, pharmacodynamics; PK, pharmacokinetics; *TREM2*, triggering receptor expressed on myeloid cells 2.

The process for developing a drug for AD and demonstrating the potential for efficacy in preclinical studies bears substantial financial burden. Moreover, to conduct translational studies in aged animal models of AD with the level of rigor required for advancing a compound to the clinic with confidence requires significant infrastructure, resources, and expertise not often accessible to academics or institutions with limited funding. To minimize this, with support from the NIA, the MODEL‐AD Preclinical Testing Core (PTC) has developed the infrastructure, resources, and validated testing pipelines for rigorous unbiased drug screening of small and large molecules that is available to the greater research community including academic, non‐profit, and for‐profit institutions with minimal costs to the investigators (Figure 2). Nominations are vetted through a web‐based portal that evaluates the drug's probability of technical success (stopadportal.synapse.org). This go/no‐go pipeline prioritizes translational outcome measures with a focus on the incorporation of rigorous best practices. The PTC pipeline includes an initial primary screen which confirms the active pharmaceutical ingredient (API) of the test compound, and evaluates drug stability, formulation, and pharmacokinetics (PK) to confirm appreciable brain exposure in the AD relevant mouse model at the pathologically relevant ages. From these measures, predictive PK/pharmacodynamics (PD) models are applied to inform the dose regimen for long‐term chronic treatment studies. The secondary screen evaluates in vivo target engagement and disease‐modifying activity using translational positron emission tomography/computed tomography, fluid biomarkers, assessment of functional activity, and potential side effects. From these PD studies, comprehensive post‐treatment multi‐omics assessment of transcriptomics and proteomics are conducted across tissues. Finally, as a tertiary screen, cognitive function is assessed using translational touchscreen‐based testing paradigms for learning, memory, and attention and correlated with neurophysiological function using in vivo electroencephalography (EEG). Essentially, this pipeline represents the lead optimization stage of compound development in which the drug candidate has been comprehensively characterized by the investigative team and is at a stage in its development and characterization that precedes the investigational new drug–enabling stage. This differs from the early target‐validation stage, when the compound is typically an initial prototype that has not yet undergone structure–activity relationship (SAR) studies to optimize central nervous system drug‐like properties, such as solubility and oral bioavailability, or to establish in vivo target engagement and pharmacokinetic/pharmacodynamic (PK/PD) relationships. These steps should be completed before the compound advances to lead optimization.

### 1.6 Remaining gaps in compound characterization that would benefit the community

In review of > 25 drug candidates that have been submitted for consideration to the Screening the Optimal Pharmaceutical for Alzheimer's Disease (STOP‐AD) portal, many of these candidates lack critical compound characterization data for advancing to lead optimization. Notably, more than half the candidates reviewed lacked selectivity data beyond their primary target including any off‐target activity to show clear demonstration that the compound's mechanism of action is specific to its primary target of action and does not have additional off‐target affinities that may have safety liabilities (e.g., Eurofins, Panlabs), and so on. Relatedly, many of the compounds also lacked in vivo PK data as well as metabolite profiling, which is critical to confirming that the compound's mechanism of action is specifically due to the parent compound and not a byproduct of biotransformation of the parent compound into metabolites that could be unrelated to the parent. While it is not necessarily cost prohibitive to generate these types of data, limited funding especially in academia and non‐profit organizations limit their prioritization. As stewards of the NIA's funding that supports the STOP‐AD portal infrastructure, it is envisioned that the pipeline would be refined to include tiered levels that allow for these additional screening activities, prior to advancing to the lead optimization‐like comprehensive in vivo characterization pipeline that has been supported by the PTC since 2016.

### 1.7 Precision medicine modeling for drug intervention studies

A major benefit of studies in animal models is the ability to investigate the trajectory of disease with incredibly high temporal resolution, prior to frank neuropathology and functional decline. This is an unrealistic scenario in human patients, especially in sporadic LOAD in which individuals often only visit their physicians when they notice *something* is wrong. Leveraging the ability to study animal models longitudinally from birth throughout their lifespan can also reveal the earliest mechanisms that drive disease inception and progression. State‐of‐the‐art multi‐omic analyses and new and expanding suites of biomarkers including proteomic‐based platforms are providing incredible insight into early molecular signatures which align with *post mortem* proteomic and/or transcriptomic signatures of *post mortem* AD brains. Therefore, instead of deciding to test the compound of interest in the model most readily available to the lab, a better precision modeling approach would be to identify the target of the drug's mechanism of action, conduct multi‐omic analyses in tissues and fluids at ages from adulthood through aging (e.g., at 2, 4, 6, 8, 12 months of age) and determine when (age point) and how robust the expression of the target protein or gene of interest is. Moreover, one can identify the model system that best recapitulates the molecular signature of the AD patient and specifically with respect to the drug target being interrogated. This can be done across model systems including as part of the comprehensive characterization of the new models being generated by MODEL‐AD. This also allows for a prophylactic approach, in which the target needs to be present to engage; but with the goal to engage as early as possible to prevent frank neuropathology and functional decline.

### 1.8 MODEL‐AD and open science

As calls for data sharing and reuse have become increasingly urgent, scientists find themselves inundated with data and the very real challenge of deciphering what is “good data.” In addition, existing research is limited by challenges in harmonizing data across different studies. In our commitment to make data openly accessible to the research community, we have established a rigorous pipeline not only for characterizing AD models, but also for sharing clean and curated data within the Findable, Accessible, Interoperable, and Reusable (FAIR) data resource the AD Knowledge Portal. As part of MODEL‐AD we have standardized data collection methods with stringent human‐led quality control that adhere to metadata standards, which is an essential element that supports reuse and allows for robust, large‐scale analyses. To date, 16 individual studies have been published on the AD Knowledge Portal. As a result, harmonized datasets from multiple studies can be combined for meta‐analysis. In addition, using standardized ‐omic data and metadata empowers researchers to derive insights with greater statistical power and meaningfully contrast the results of studies using different models in apples‐to‐apples comparisons, potentially advancing precision medicine for AD. As artificial intelligence development continues to move rapidly, access to real, curated, high‐quality datasets is essential for model evaluation. Within the MODEL‐AD consortium, we are doing science with the structure and transparency needed to make the data and the results trustworthy.

## 2 CONCLUSION

Over the past decade, MODEL‐AD has transformed the landscape of preclinical AD research by establishing genetically relevant models, rigorous phenotyping pipelines, and open‐access resources aligned with human pathology and precision medicine approaches. As our understanding of LOAD continues to evolve, acknowledging its complex, multifactorial, and age‐associated nature, so too must our models and methodologies. MODEL‐AD is uniquely positioned to lead this next phase by integrating genetic, environmental, vascular, and co‐pathological contributors into a unified translational framework. Through close collaboration with other consortia, deployment of standardized data pipelines, and a commitment to transparency and open science, the program offers an unprecedented foundation for mechanistic discovery and therapeutic advancement. Continued investment in MODEL‐AD will not only accelerate the development of effective interventions for LOAD and related dementias but will also serve as a scalable model for future efforts in age‐related neurodegenerative disease research.

In our commitment to make data openly accessible to the research community, we have established a rigorous pipeline not only for characterizing AD models, but also for sharing clean and curated data within the FAIR data resource in the AD Knowledge Portal (PMID: 33085189).

## CONFLICT OF INTEREST STATEMENT

Adrian L. Oblak reports research support from the NIH and Indiana University School of Medicine, research funding from Monument Biosciences, honoraria from the American Academy of Geriatric Psychiatrists and Eli Lilly, and travel support from AAIC and the University of California Irvine. Bruce T. Lamb reports support from the NIH, Indiana University School of Medicine, and Indiana University Health; royalties from Ionis Pharmaceuticals; consulting and advisory roles with NervGen Inc., Cleveland Clinic, and UCLA; leadership roles with the Alzheimer's Association and Cure Alzheimer's Fund; and stock options in Monument Biosciences. Michael Sasner reports NIH grant support, royalties and patent filings through The Jackson Laboratory, NIH honoraria, and conference travel support from the International Neuroimmune Consortium. Paul R. Territo reports NIH/NIA grant support and service on scientific advisory boards for MODEL‐AD, ADCS, and Can Thumbs Up. Gregory W. Carter reports NIH and Department of Defense grant support, royalties and patent filings through The Jackson Laboratory, consulting for Astrex Pharmaceuticals, honoraria, and conference travel support. Stacey J. Sukoff Rizzo reports NIH/NIA and foundation grant support; consulting for Hager Biosciences and Genprex Inc.; lecture honoraria, travel support, advisory and leadership roles, stock ownership in multiple companies including Monument Biosciences; and an adjunct faculty appointment at The Jackson Laboratory. Gareth Howell reports NIH grant support, royalties and patent filings through The Jackson Laboratory, and NIH honoraria. Karina Leal declares no conflicts of interest. Author disclosures are available in the Supporting Information.

## Supporting information



Supporting Information
